# Prevalence and Treatment Outcome of Displaced High-Long Oblique Supracondylar Humeral Fractures in Children

**DOI:** 10.3389/fped.2021.739909

**Published:** 2021-10-27

**Authors:** Mudit Shah, Joo Hyung Han, Hoon Park, Hyun Woo Kim, Kun-Bo Park

**Affiliations:** ^1^Division of Pediatric Orthopedic Surgery, Severance Children's Hospital, Yonsei University College of Medicine, Seoul, South Korea; ^2^Department of Orthopedics, Gangnam Severance Hospital, Yonsei University College of Medicine, Seoul, South Korea

**Keywords:** humerus, supracondylar fracture, long oblique fracture, percutaneous pinning, children

## Abstract

**Aim:** The treatment protocol for supracondylar humeral fracture has mainly been based only on the severity of displacement and percutaneous pinning has been recommend as a first treatment. However, a long oblique fracture line is difficult to fix by the traditional cross pinning. The purpose of this study is to assess the prevalence of high-long oblique supracondylar humeral (HLO) fracture and evaluate the surgical outcome of percutaneous pin fixation.

**Methods:** We reviewed 690 children who had undergone an operation for the displaced supracondylar humeral fracture. HLO fracture was defined as having a fracture line starting from either cortex above the metaphyseal-diaphyseal junction and finishing at the opposite cortex around or below the olecranon fossa. Clinical and radiographic parameter outcomes were assessed.

**Results:** There were 14 patients diagnosed with the HLO fracture (14/690) and all the patients were treated by pin fixation. The median age was 5 years 1 month (range, 2–11 years). The common mode of injury was direct contact injury to the elbow. There were 6 patients with lateral HLO fracture, and 8 patients had medial HLO type. In medial HLO type, medial pinning only was done in 3 patients due to the difficulty in lateral pin insertion. In addition, the lateral pin was not a bicortical fixation through capitellum entry in 2 patients who had it fixed by cross pinning. The final Baumann angle and lateral humero-capitellar angle were 20.5 (5–67.6) degrees and 49.3 (23.3–71.9) degrees, respectively, without statistical significance compared to the normal side. Flynn's cosmetic grade showed satisfactory results in all patients.

**Conclusion:** The prevalence of HLO fractures was 2% in the displaced supracondylar humeral fracture. The mechanism of injury of HLO fractures may be direct contact injury. In medial HLO fractures, medial pinning is important for stability, and sometimes lateral pinning was impossible. Contrarily, lateral HLO fracture could easily be fixed by lateral-only pinning, but the correct lateral pinning is necessary because medial pinning is difficult. The HLO fracture is a difficult pattern to treat by traditional percutaneous pinning and another surgical option should be considered.

## Introduction

Supracondylar humeral fracture is the most common elbow fracture in children, and the treatment protocol has mainly been based on the severity of displacement (1–3). Historically the displaced supracondylar fracture (Gartland type III or IV) has been defined as a fracture of the metaphyseal region of the distal humerus with the fracture line crossing both medial and lateral columns without involving the intercondylar region, and numerous orthopedic surgeons still believe that the appropriate treatment for the displaced pediatric supracondylar humeral fracture would be percutaneous pinning and cast immobilization after closed or open reduction.

However, all the supracondylar humeral fractures are not transverse types (AO 13-M/3.1 III and IV, 13-M/3.2 III and IV) and other surgical options could be more appropriate (4–10). Bahk et al. classified the high and oblique type of supracondylar humeral fracture because of fixation difficulty (4). The distal humerus metaphyseal-diaphyseal junction (MDJ) type fracture has also been described (5, 7). Elastic stable intramedullary nailing for high supracondylar MDJ fractures have been reported due to the difficulty in achieving bicortical fixation with Kirschner wires (K-wires) (5, 9–11). Lateral external fixator, another surgical option, has also demonstrated superior outcome for oblique or comminuted fracture (6). The traditional percutaneous pinning for the pediatric supracondylar fracture should be modified based on more precise classification and diverse treatment methods, not just on the severity of displacement (4, 8, 9).

We experienced a high-long oblique type of supracondylar humeral (HLO) fracture involving and extending beyond the supracondylar and MDJ region where fixation with traditional cross-pinning was technically challenging. The purpose of this study was (1) to assess the prevalence of displaced HLO supracondylar humeral fracture and (2) to evaluate the surgical outcome of pin fixation for the HLO supracondylar humeral fracture in children.

## Patients And Methods

This study was a retrospective cohort study and approved by our institutional review board (4-2017-0210). Between January 2000 and June 2020, we reviewed 719 children with supracondylar humeral fractures operated at a single tertiary pediatric care hospital.

**Figure 1:**
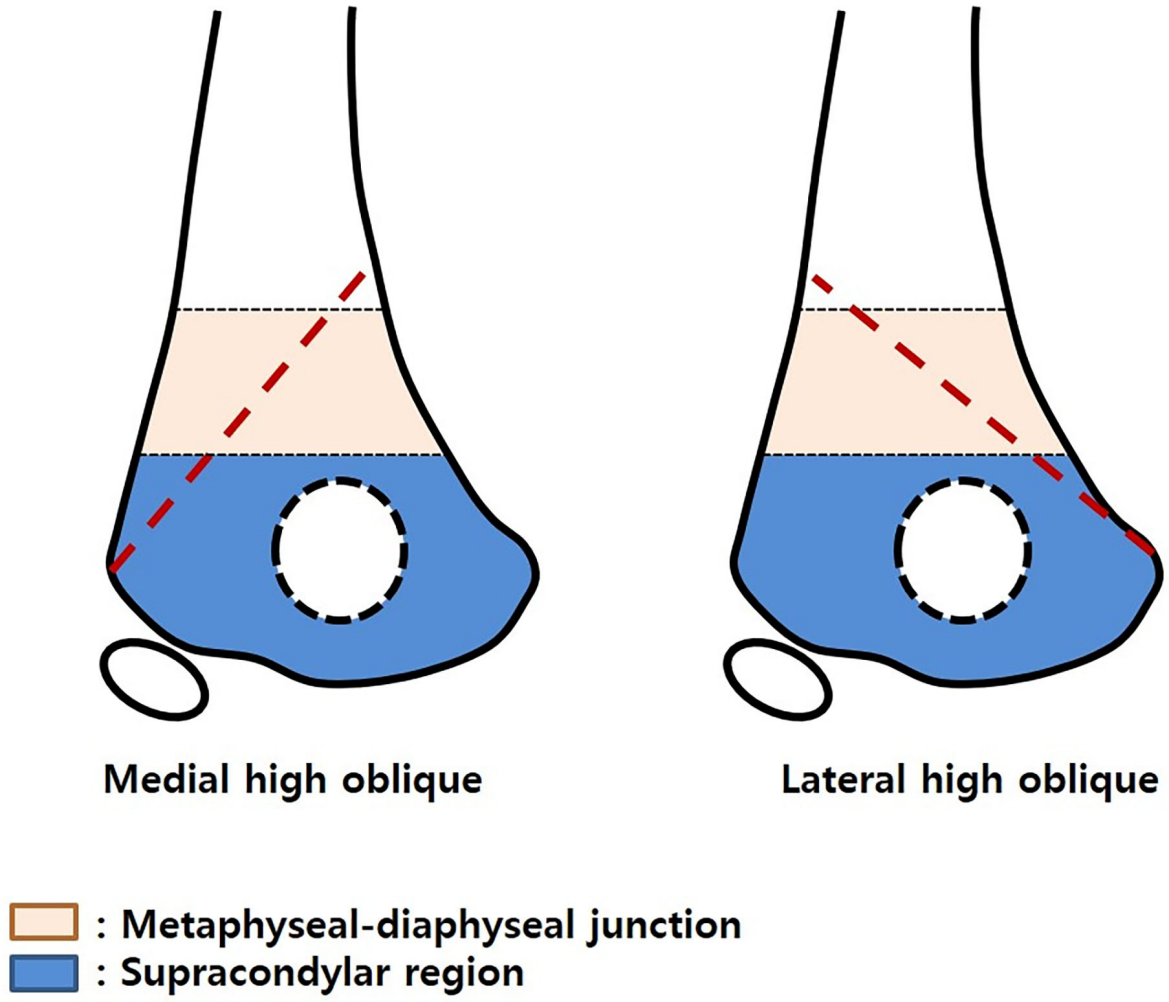
Illustration of the high-long oblique supracondylar humeral fracture.

Inclusion criteria were patients with (1) displaced supracondylar humeral fracture and (2) age <12 years old. Exclusion criteria were (1) children with other bone diseases, such as osteogenesis imperfecta or rickets, (2) repeat supracondylar humeral fracture, (3) fracture in children with neuromuscular problems, and (4) fracture in children with a congenital deformity of the upper extremity. There were 690 patients who met the inclusion/exclusion criteria. HLO fracture was defined as having a fracture line starting from either cortex above the metaphyseal-diaphyseal junction and finishing at the opposite cortex around or below the olecranon fossa (Figure 1).

Clinical details and operative records were available through the electronic medical records for each patient. All fractures were fixed by closed/open reduction and K-wires. After fixation, they were kept in a long-arm cast for 4 weeks. After 4 weeks, the wires were removed if the fracture was healed. If the fracture healing looked incomplete, a removable splint was prescribed for 1 week. The child was encouraged to engage in active elbow range of motion (ROM) exercises. After 4 weeks, the child was followed up to check the elbow ROM. An additional follow-up was recommended to patients who did not have full ROM or had any other complaint after 4 weeks.

Standard anteroposterior (AP) and lateral radiographs were used to assess the pattern of fracture at the time of injury. Baumann angle and lateral humero-capitellar angle were assessed on elbow X-rays at the most recent follow-up. Baumann's angle was measured on the AP radiograph of the elbow by drawing a line perpendicular to the longitudinal axis of the humeral shaft and a line following the physeal line of lateral condyle (12). The angle between the humeral shaft and the capitellum on a lateral radiograph was defined as the lateral humero-capitellar angle (4). The malrotation was defined as a difference of more than 20% between the width of the distal humerus above and below the fracture site on a lateral radiograph (4). In the HLO fracture, the mid-point of the fracture line was taken as the reference point when assessing malrotation. The cosmetic and functional outcome was evaluated at the final follow-up using the Flynn classification (13).

Statistical analyses were performed using the SPSS v.23 software (IBM, Armonk, New York). The Mann-Whitney U test was used to compare the final follow-up radiographs with the radiographs of the normal side. The results were presented as the median value (range, min-max value). The level of significance was set at *p* < 0.05.

## Results

**Figure 2:**
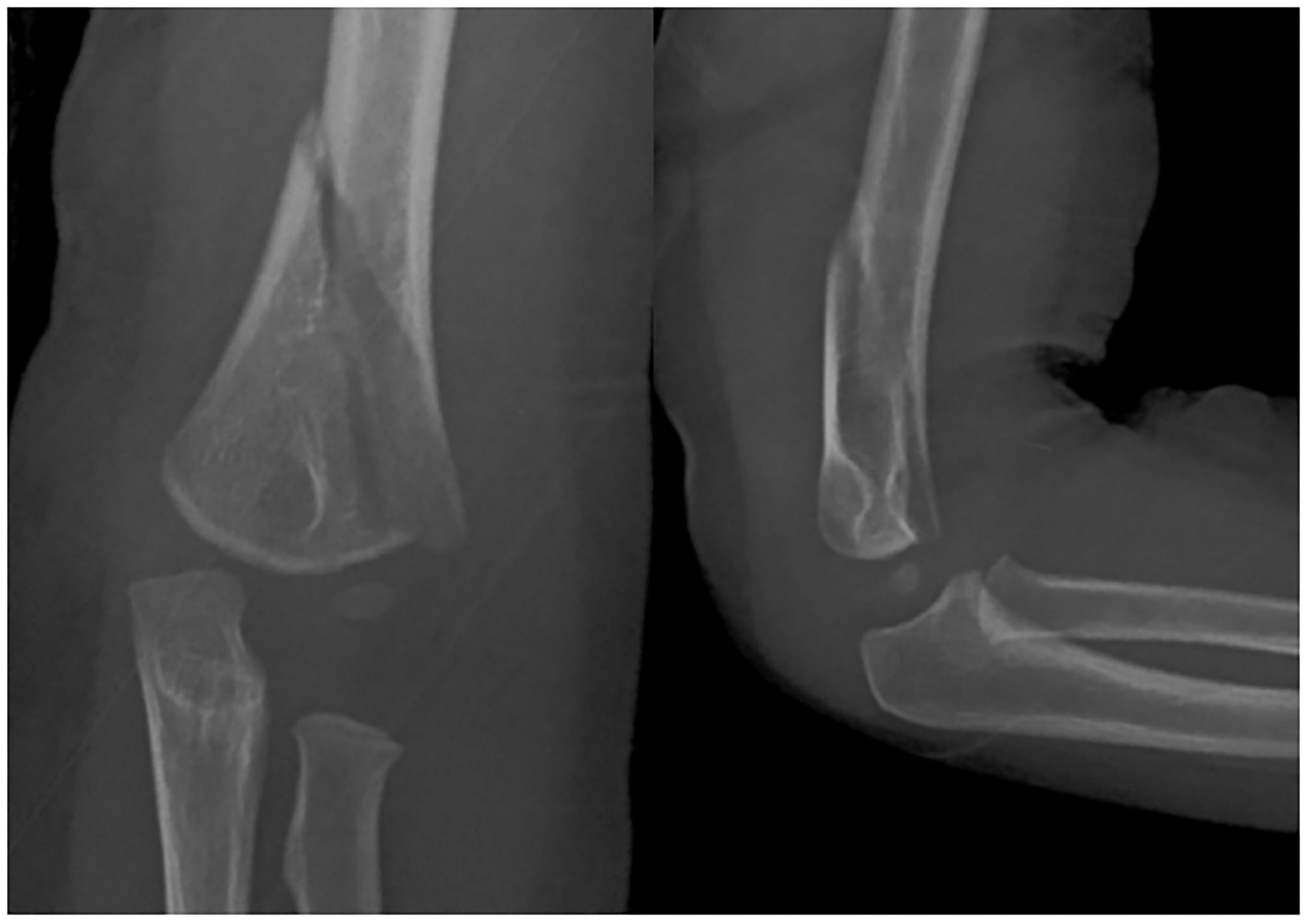
A 2-year-old child fell from the bed and had a direct contact injury to the left elbow. The radiographs demonstrate a completely displaced medial high-long oblique supracondylar humeral fracture.

The prevalence of displaced HLO supracondylar humeral fracture was 2.0% (14/690) (Table 1). The median age of patients was 5 years 1 month (range, 2–11 years) at the time of diagnosis. There were 12 boys and 2 girls. Ten patients had the fracture on the left arm, and four patients had the fracture on the right side. The mean body mass index (BMI) was 15.3 kg/m^2^ (range, 13.3–18.7 kg/m^2^). The most common mode of injury was a direct contact injury to the elbow in 13 out of 14 patients (Figure 2). These direct injuries occurred when the child fell from a height of 1–1.8 m and landed directly on the elbow, as witnessed by caregivers. Only one child had an injury in the classical way of fall on an outstretched hand (FOOSH). The mean follow-up period was 6 months (range, 2–17 months). All fractures were closed fractures with only one case showing superficial abrasion, which resolved spontaneously. None of the patients had compartment syndrome, neurovascular injury, or any other injury in the ipsilateral limb.

**Table 1:** Patient demographics.

**No**.	**Age (year)**	**Sex**	**BMI (kg/m^**2**^)**	**Side**	**Fracture type**	**Mode of injury**	**Reduction**	**K-wires**	**Fixation problem**
1	2	M	15.5	Lt	Lateral HLO	Direct contact (1 m)	Closed	2 (2 lat)	Non-capitellar entry
2	6	M	13.6	Lt	Lateral HLO	Direct contact (1 m)	Closed	5 (4 lat, 1 med)	
3	4	M	14.7	Lt	Lateral HLO	Direct contact (1 m)	Closed	3 (3 lat)	
4	5	M	16.8	Rt	Lateral HLO	Direct contact (1.5 m)	Closed	3 (3 lat)	
5	6	M	14.1	Lt	Lateral HLO	Direct contact (1 m)	Closed	3 (3 lat)	
6	6	M	18.7	Rt	Lateral HLO	Direct contact (1 m)	Closed	2 (2 lat)	Non-capitellar entry
7	7	M	13.3	Lt	Medial HLO	Direct contact (1 m)	Open	3 (2 lat, 1 med)	
8	11	M	15.9	Lt	Medial HLO	Direct contact (1 m)	Closed	4 (2 lat, 2 med)	
9	2	M	14.2	Lt	Medial HLO	Direct contact (0.5 m)	Closed	3 (3 med)	
10	2	M	14.4	Lt	Medial HLO	Direct contact (1 m)	Closed	3 (2 lat, 1 med)	
11	7	M	16.3	Rt	Medial HLO	Direct contact (1.8 m)	Closed	4 (2 lat, 2 med)	
12	2	F	15.7	Lt	Medial HLO	Direct contact (1 m)	Closed	2 (2 med)	Intramedullary pinning
13	8	F	15.1	Rt	Medial HLO	FOOSH	Closed	3 (3 med)	Intramedullary pinning
14	3	M	16.1	Lt	Medial HLO	Direct contact (1 m)	Closed	3 (2 lat, 1 med)	

*M, male; F, female; HLO, high-long oblique; FOOSH, fall on outstretched hand; K-wire, Kirschner wire; lat, lateral; med, medial*.

**Figure 3:**
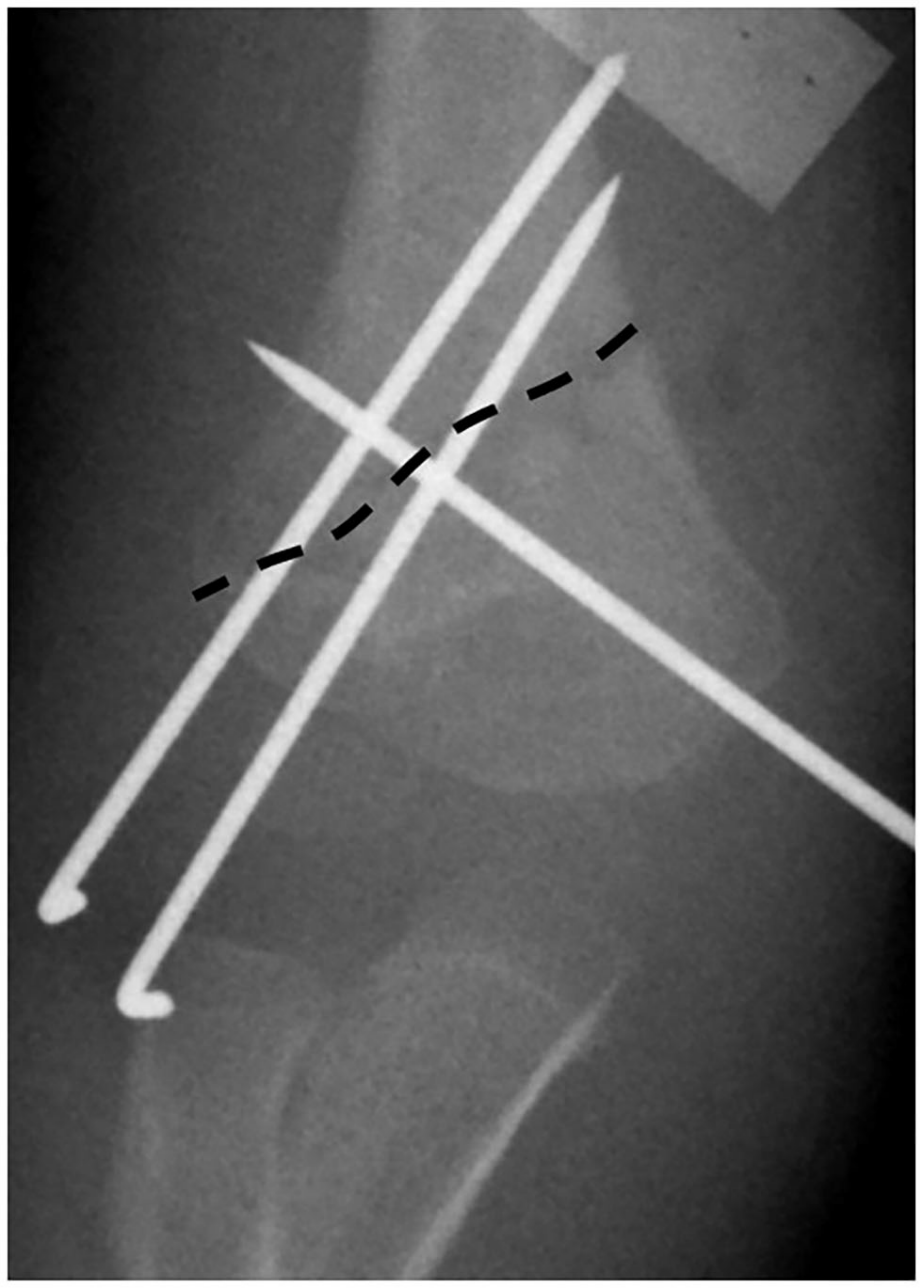
The dashed line shows the fracture line. Cross-pin fixation was possible in this medial high-long oblique supracondylar humeral fracture; however, one lateral wire could not get the capitellar entry. Another pin was convergent due to the medial high fracture line.

**Figure 4:**
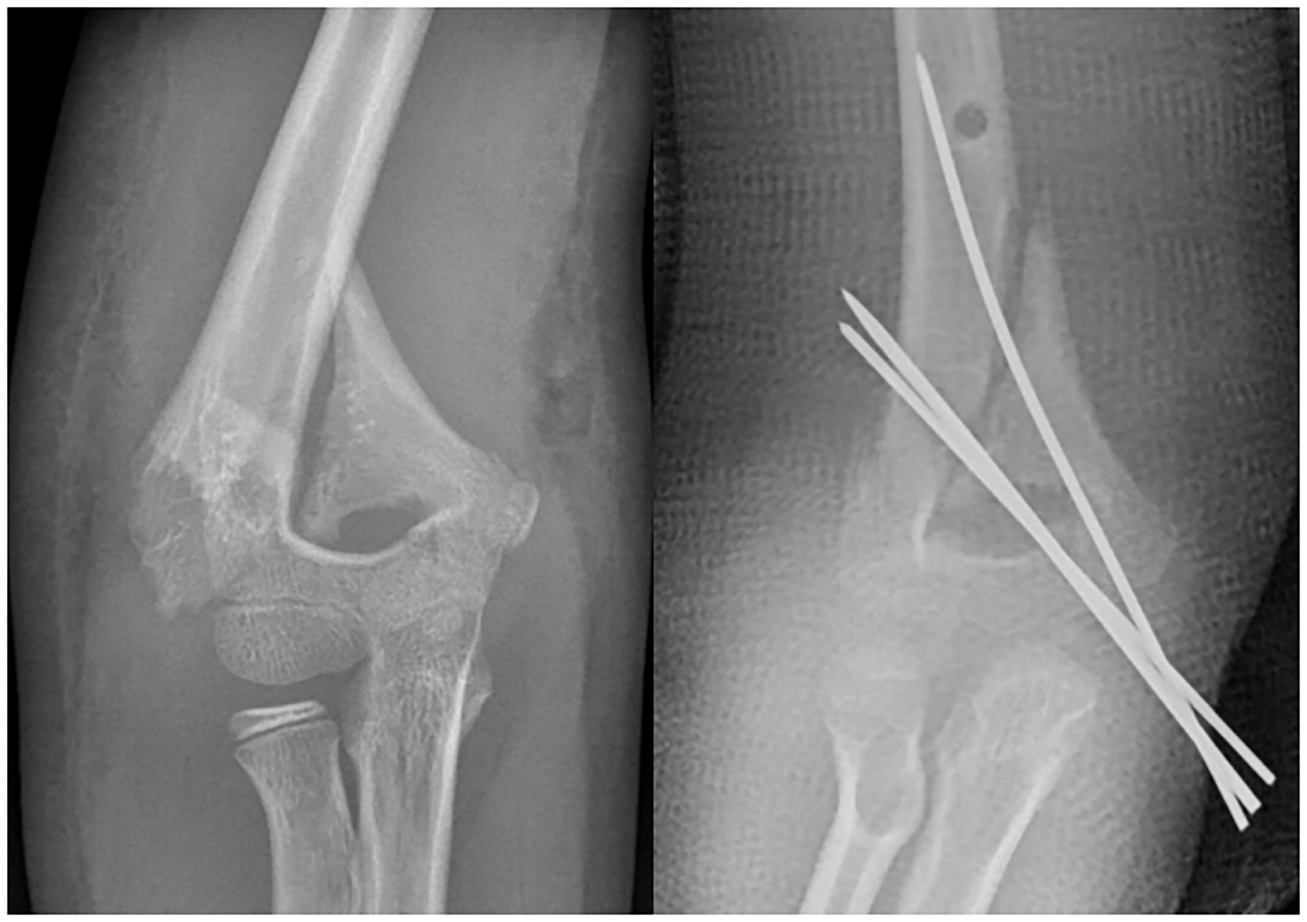
The lateral pin fixation was impossible in this medial high-long oblique supracondylar humeral fracture. Multiple medial pin insertion was done with medial incision and ulnar nerve protection.

In our cohort of patients, there were 6 (6/690) patients with lateral HLO fracture. All these fractures were treated by closed reduction and K-wires. There were 5/6 treated by lateral pinning only, but non-capitellar entry due to the obliquity of the fracture line was seen in 2/6 of the fractures. There were 8 (8/690) patients with medial HLO fractures. There were7 (7/8) fractures fixed with closed reduction and K-wires, but 1 (1/8) patient had to be managed by open reduction and K-wire fixation. Medial pinning only was done in 3/8 patients due to difficulty in lateral pin insertion, but in 5/8 patients, cross-pin fixation was done (Figure 3). However, in 2 patients, the surgeon failed to achieve bicortical fixation and had an intramedullary pin in these cases (Figure 4). Flynn's cosmetic grade showed satisfactory results in all patients. Flynn's functional grade showed satisfactory results in 12 patients and unsatisfactory results in 2 patients with a mild flexion deformity (Table 2). The post-operative casting period was relatively uneventful in all patients.

**Table 2:** Clinical results (according to Flynn's criteria) for both fracture subtypes.

**Type**	**Results**	**Number of patients**	**Percentage (%)**
Lateral high-long oblique	Excellent	5	83.3
	Good	0	0
	Fair	0	0
	Poor	1	16.7
Medial high-long oblique	Excellent	7	87.5
	Good	0	0
	Fair	0	0
	Poor	1	12.5

There was no statistically significant difference in the Baumann angle at the final follow-up 20.5 (5–67.6) degrees compared to the normal side 20.5 (11.2–29.5) degrees (*p* = 0.275). There was no statistically significant difference between the final humero-capitellar angle 49.3 (23.3–71.9) degrees and normal humero-capitellar angle 42.9 (27.6–67.9) degrees (*p* = 0.169; Table 3). Malrotation was seen in two patients, one in each of medial and lateral HLO fractures, respectively. However, these patients did not show any significant cubitus varus or limitation of motion at the final follow-up.

**Table 3:** Radiological parameters for both fracture subtypes.

**Type**	**Parameters**	**Normal side**	**Final affected side**	***P*-value**
Lateral high-long oblique	Baumann angle	19.1 (11.2–29.5)	24.8 (14–67.6)	0.772
	Lateral humero-capitellar angle	41.7 (32.7–67.9)	49.8 (23.3–71.9)	0.189
Medial high-long oblique	Baumann angle	22.0 (15.5–27.3)	14.6 (5–34)	0.093
	Lateral humero-capitellar angle	44.3 (27.6–57.9)	48.7 (32.6–59.2)	0.485

## Discussion

The Gartland classification mainly described the severity of displacement in the coronal and sagittal planes (1, 14–16). However, Gartland classification does not consider the fracture pattern, such as level of fracture, obliquity of the fracture line, and comminution of fracture. Sen et al. have mentioned the role of conservative treatment of the oblique and comminuted type of MDJ fractures due to the high remodeling potential of the metaphyseal fracture and increased area of contact, but they have not described the surgical treatment of the unstable fracture types (7). Bahk et al. described a subtype of coronal and sagittal fracture line with a >10-degree obliquity and a high fracture pattern. The high type which Bahk et al. mentioned was challenging to manage with percutaneous pinning; however, they did not mention the obliquity in the high-type fracture (4). AO classification of the supracondylar humeral fracture is more precise with a wide acceptance and high reliability (17). Furthermore, external fixation was recommended as an alternative technique for the patterns that are difficult to stabilize with K-wires. We introduce this displaced HLO supracondylar humeral fracture as a difficult pattern to treat with traditional percutaneous cross-pin fixation and surgeons should consider other surgical options.

The displaced HLO supracondylar humeral fracture is a rare type of fracture with a prevalence of only 2% in this study. The mechanism of injury of this fracture may be different compared to the transverse supracondylar humeral fracture or the MDJ fracture pattern. Almost all except one patient had a direct injury to the elbow, which is contrary to the well-known mechanism of fall on outstretched hand for extension type of supracondylar humerus fractures. The median age at operation was 5 years, but it is hard to say this fracture is common in younger children because the age range was 2–11 years and the study cohort had only 14 patients.

The lateral pin fixation for medial HLO fracture is complex because the fracture line corresponds with the line of pin insertion. There was a need for definite medial pin fixation for these fractures. For safe medial pin fixation, the position of the elbow should be extended to prevent injury to the ulnar nerve after lateral pin fixation. However, under the unstable lateral pin fixation, elbow extension may give rise to the loss of reduction. In our experience, sometimes the medial pin was fixed first, and the lateral pin was followed if cross-pinning was done. In all cases, a medial incision was needed to prevent ulnar nerve injury. Furthermore, lateral pin fixation was impossible in three patients, and so additional bicortical medial pin fixation was better than lateral medullary pin fixation. In our cohort of patients, two patients had intra-medullary pins. Fortunately, the clinical and radiographic outcomes of these patients were acceptable, but we do not recommend intramedullary fixation because of the weakness against the torsional force. Instead, if the radial external fixator is used before or after medial pin fixation (9), there would be powerful stability. We did not have experience using an external fixator because the external fixator for the upper extremity fracture was not common during this study period. However, surgeons may expect a predictable outcome with the external fixator in this technically demanding fracture for only pin fixation.

**Figure 5:**
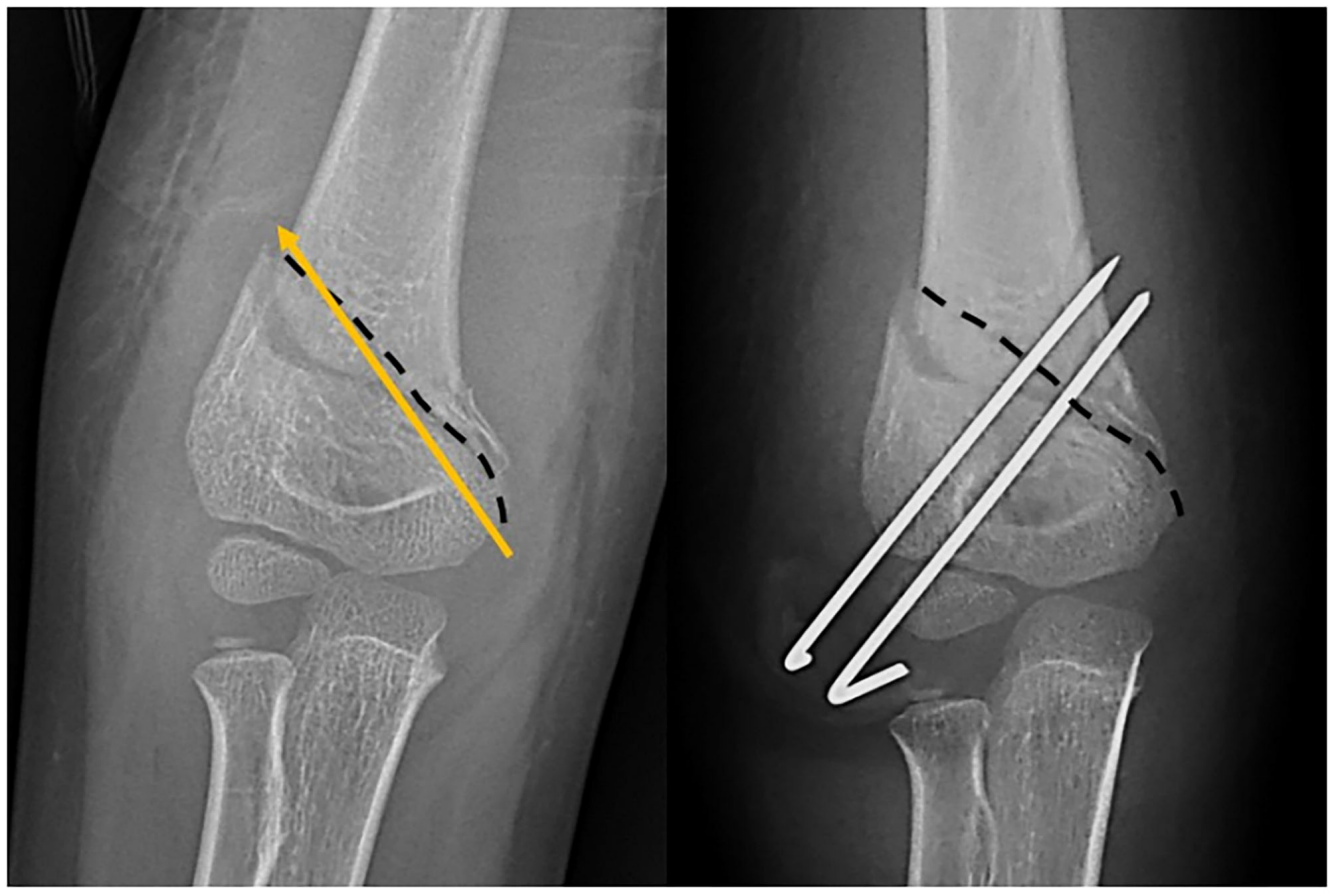
The dashed line demonstrates the lateral high-long oblique fracture. The arrow shows that the medial wire cannot insert in these kinds of fracture patterns.

Compared to the medial HLO subtype, fixation of the lateral HLO type was relatively more straightforward (Figure 5). Although medial pinning is problematic because of the pattern of the fracture line, they were fixed with lateral pinning in 5/6 cases, and cross-pinning was done in 1/6 patients. However, the authors would like to mention that due to the long obliquity of the fracture line, it is sometimes not possible to gain capitellar entry. Capitellar entry has been shown to provide maximum torsional resistance allowing the surgeon to gain sufficient bone in the distal fragment and provide maximum wire separation at the fracture site (11). Due to the long obliquity of the fracture plane, K-wires were inserted through the non-capitellar lateral cortex of the distal fragment in two cases. In our opinion, an external fixator could also be helpful for the additional stability in lateral HLO fracture. However, the elastic stable intramedullary nail only may not be appropriate because of the obliquity and distal extension of the fracture line (11).

Previous literature corroborates that high supracondylar humeral fracture makes it difficult to place two or three bicortical lateral pins and achieve adequate proximal medial column purchase, especially in a small child (15, 18). MDJ fractures are determined by the square described in the AO pediatric fracture classification (17). At MDJ, the cortical bone is beginning, and the insertion angle is too flat. These two reasons make stable K-wire fixation difficult. The most common reason for loss of pin fixation in the displaced supracondylar humeral fracture is usually due to technical problems, such as sub-optimal pin configuration and failure to achieve bicortical fixation (19, 20). Due to the obliquity of the fracture line, bicortical lateral pin fixation is very difficult in medial HLO. For the lateral HLO, the surgeon could not fix a medial pin, so wide separation of the lateral inserted K-wires at the fracture site is necessary (19, 21). The unstable fixation may result in fixation loss or malrotation, and malrotation is an important factor associated with developing the cubitus varus (22, 23). Two patients in our study, one from each subgroup of medial HLO and lateral HLO fractures, showed malrotation on lateral x-rays but there was no clinically significant deformity seen at the latest follow-up.

This study has several limitations. We have included only the displaced type of HLO fracture subtype. The undisplaced fractures are treated on an outpatient basis. If we include conservative cases, the prevalence may be different. Due to the low prevalence of the fracture type described in this study, the number of patients was small. To understand the mechanism of this rare fracture pattern, a multi-center study with more cases or biomechanical research should be followed. Even though our results were acceptable in terms of no cubitus varus and no ulnar nerve injury, the trouble of fixation cannot be underestimated in these fractures. Finding the most appropriate treatment for this kind of fracture pattern remains a challenge. Although we have no experience about the use of external fixator for supracondylar humeral fracture, radial external fixator can be utilized as one of the treatment modalities for this kind of difficult SCH fracture fixation (9). A prospective study with supported biomechanical assessment should be considered in future research.

The prevalence of displaced HLO type SCH fractures in children is 2%, and HLO fractures need to be distinguished from the typical SCH fractures. The mechanism of injury of HLO fractures may be direct contact injury to the elbow. Medial HLO fractures can be treated with cross-pin fixation if the surgeon is able to insert the bicortical lateral wire successfully. However, stable lateral pin insertion is usually difficult, and medial pin fixation is more critical in terms of stability. Sometimes, multiple medial pinning is the only surgical fixation method with a medial incision and ulnar nerve protection. In our opinion, percutaneous pinning and cast immobilization, especially lateral only pinning, would be a relative contraindication for medial HLO fractures. It is challenging to perform the cross-pin fixation in lateral HLO fractures, and thus the surgeon should aim for the correct lateral only pinning to maximize fracture stability. To increase stability during or after operation for this HLO fracture, surgeons should consider another surgical option beyond percutaneous pinning, such as external fixator.

## Data Availability Statement

The original contributions presented in the study are included in the article/supplementary material, further inquiries can be directed to the corresponding author/s.

## Ethics Statement

The studies involving human participants were reviewed and approved by Yonsei University Health System, Severance Hospital, Institutional Review Board. Written informed consent from the participants' legal guardian/next of kin was not required to participate in this study in accordance with the national legislation and the institutional requirements.

## Author Contributions

MS, JH, and HP contributed to data analysis. HK contributed to supervision. MS and K-BP drafted the manuscript. MS, HP, HK, and K-BP edited and revised the manuscript. All authors have read the manuscript, agreed to its being submitted for publication, contributed to the conception, and design of the research.

## Funding

This research was supported by Mid-Career Research Program through the National Research Foundation of Korea (NRF) funded by the Ministry of Education (2020R1A2C1006454).

## Conflict of Interest

The authors declare that the research was conducted in the absence of any commercial or financial relationships that could be construed as a potential conflict of interest.

## Publisher's Note

All claims expressed in this article are solely those of the authors and do not necessarily represent those of their affiliated organizations, or those of the publisher, the editors and the reviewers. Any product that may be evaluated in this article, or claim that may be made by its manufacturer, is not guaranteed or endorsed by the publisher.
